# Mesenchymal stem cells improve liver fibrosis and protect hepatocytes by promoting microRNA-148a-5p-mediated inhibition of Notch signaling pathway

**DOI:** 10.1186/s13287-022-03030-8

**Published:** 2022-07-26

**Authors:** Qing Zhou, Chao Rong, Tengfei Gu, Hongda Li, Lei Wu, Xuemei Zhuansun, Xin Zhao, Zuorun Xiao, Yuting Kuang, Sanrong Xu, Shouli Wang

**Affiliations:** 1 Department of Pathology, School of Biology and Basic Medical Sciences, Suzhou Medical College, Soochow University, Suzhou, 215123 China; 2 Department of General Surgery, Affiliated Hospital of Jiangsu University, Zhenjiang, 212001 China; 3 Department of Anesthesiology, People’s Hospital of Lianshui County, Huaian, 223400 China; 4 Department of General Surgery, First Affiliated Hospital of Soochow University, Suzhou, 215008 China

**Keywords:** Mesenchymal stem cells, Hepatic stellate cell, MicroRNA-148a-5p, Liver fibrosis, Notch2, Notch signaling pathway

## Abstract

**Background:**

Mesenchymal stem cells (MSCs) are considered to be a potential therapeutic tool for liver fibrosis. Inhibiting the activation of hepatic stellate cells (HSCs) and protecting hepatocytes are important mechanisms for the anti-fibrotic effect of MSCs. However, how MSCs inhibit liver fibrosis by regulating the expression of microRNAs (miRNAs) has not been fully clarified.

**Methods:**

Transforming growth factor-β1 (TGF-β1)-activated HSCs LX-2 were single cultured or co-cultured with human umbilical cord mesenchymal stem cells (HUC-MSCs). High-throughput sequencing was used to evaluate the differentially expressed microRNAs (DEMs) between the two groups. Quantitative real-time PCR (qRT-PCR), Western blot, and transfection experiments were used to investigate and screen the most significantly up-regulated DEM. Bioinformatics analysis was used to predict the target mRNAs and the potential functions of the DEM. The possible mechanism of HUC-MSCs against liver fibrosis was analyzed by co-culture experiment of HUC-MSCs with LX-2 cells, and HUC-MSCs treatment of Bile duct ligation (BDL)-induced liver fibrosis in mice. Finally, the mechanism of the DEM regulating liver fibrosis was confirmed in human liver fibrosis specimens.

**Results:**

MicroRNA-148a-5p (miR-148a-5p) was the most significantly up-regulated DEM in activated LX-2 cells co-cultured with HUC-MSCs compared with LX-2 cells single cultured. Up-regulation of the expression of miR-148a-5p in activated LX-2 cells could significantly inhibit the expression of hepatic fibrosis markers α-SMA and Col1α1. Notch2 was one target gene of miR-148a-5p. Co-cultured with HUC-MSCs could inhibit the activation of LX-2 cells by inhibiting the expression of the Notch2 and the Notch signaling pathway. In addition, HUC-MSCs treatment could up-regulate the expression of miR-148a-5p in liver tissue and hepatocytes, promote the proliferation and avoid the apoptosis of hepatocytes, and reduce the degree of fibrosis by inhibiting expression of the Notch2 and the Notch signaling pathway in BDL-induced liver fibrosis mice. Moreover, miR-148a-5p was down-regulated and Notch2 was up-regulated in fibrotic human liver tissues compared with the normal livers.

**Conclusions:**

HUC-MSCs treatment could inhibit HSCs activation, protect hepatocytes, and alleviate BDL-induced liver fibrosis in mice by up-regulating the expression of miR-148-5p and inhibiting the Notch signaling pathway. The down-regulation of miR-148-5p and up-regulation of Notch2 could be used as biomarkers to monitor the progression of liver fibrosis.

**Supplementary Information:**

The online version contains supplementary material available at 10.1186/s13287-022-03030-8.

## Introduction

Liver fibrosis is a common pathological process of various types of chronic liver diseases, which eventually develop into cirrhosis, and it is characterized by the gradual replacement of functional liver tissue by extracellular matrix (ECM) [1]. Normally, hepatic stellate cells (HSCs) maintain nonproliferative status and function as vitamin A-storing cells in the normal liver [2]. Upon liver injury, HSCs are activated and characterized by proliferative, contractile, and myofibroblastic features, and serve as the main ECM-producing cells which contribute to liver fibrosis [3, 4]. Therefore, regulating of HSCs activation could be a potential antifibrotic strategy [5].

Mesenchymal stem cells (MSCs)-based therapy may be a potential therapeutic method for liver fibrosis [6, 7]. MSCs have been successfully isolated from a variety of tissues such as bone marrow, adipose, placenta, umbilical cord, and umbilical cord blood [8, 9] and identified by spindle-shape appearance, classical three-lineage differentiation abilities, and surface markers’ expression [10, 11]. In recent years, accumulated studies have shown that MSCs can inhibit the activation of HSCs and lessen hepatic fibrosis in experimental animals [12–14]. In clinical studies, MSCs can also promote the recovery of liver function and liver regeneration [15, 16]. Human umbilical cord mesenchymal stem cells (HUC-MSCs) are usually isolated from the Wharton’s Jelly of umbilical cord. Because of their easily been obtained, no ethical concern, and low immunogenicity for major histocompatibility complex class I (MHCI) dull and negative for MHC class II (MHCII), HUC-MSCs are often used as allogeneic cells for tissue repair and do not induce observable immune responses [17]. It has been reported that HUC-MSCs transplantation can promote the recovery of liver function in rats with acute liver failure [18], and peripheral infusion of HUC-MSCs can rescue acute liver failure lethality in monkeys [19].

MicroRNAs (miRNAs) are a type of nonencoding small RNA with a length of 18–22 nucleotides and can regulate protein expression at the mRNA level [20]. Recent studies have shown that the activation of HSCs and progression of liver fibrosis are accompanied by differential expression of miRNAs, suggesting that miRNAs may play an important role in regulating various biological processes associated with liver fibrosis [21, 22]. However, the underlying mechanism of MSCs alleviating liver fibrosis by regulating miRNAs expression remains unclear.

miR-148a is frequently dysregulated in various liver diseases, such as hepatocellular carcinoma (HCC), alcoholic hepatitis, abnormal hepatic lipid metabolism, and liver fibrosis [23–25]. Down-regulation of miR-148a in HCC is often associated with poor prognosis [26]. Overexpressing of miR-148a can inhibit cell proliferation, induce autophagy and promote apoptosis of HSCs [27].

In the present study, we have found that miR-148a-5p was significantly up-regulated in TGF-β1-activated HSCs after co-cultured with HUC-MSCs by high-throughput sequencing, and HUC-MSCs treatment could significantly ameliorate HSCs activation by up-regulating miR-148a-5p and inhibiting the expression of its target gene Notch2 in vitro. In vivo, we have also showed that HUC-MSCs could up-regulate miR-148a-5p expression in liver tissue and relieve liver fibrosis in mice by inhibiting the Notch signaling pathway.

## Materials and methods

### Isolation and culture of HUC-MSCs

Human umbilical cords were freshly obtained from mothers who underwent caesarean section in the Affiliated Hospital of Jiangsu University (Zhenjiang, China) and rapidly processed. All the donors had been informed and provided a written informed consent. The protocols were approved by the Scientific Research Ethics Committee of Affiliated Hospital of Jiangsu University (Zhenjiang, China). HUC-MSCs were isolated as described previously [28]. HUC-MSCs were cultured in low glucose Dulbecco’s modified Eagle’s medium (DMEM-LG, Gibco, San Diego, CA, USA) supplemented with 10% fetal bovine serum (FBS, Gibco, Grand Island, NY, USA) and 100 IU/mL penicillin/streptomycin (Gibco) in a humidified atmosphere containing 5% CO_2_ at 37 °C, and the medium was changed every 2–3 days. Cells were detached by using 0.25% trypsin–EDTA (Thermo Fisher, Waltham, MA, USA) upon reaching 80% confluence. The capacity of differentiation into adipocytes, osteoblasts, and chondrocytes, and the surface antigen clusters of HUC-MSCs were detected by FACS as previously reported [28, 29].

### HSC culture and activated by TGF-β1

Human immortalized HSC line LX-2 cells were purchased from Shanghai Biological Technology Co., Ltd. enzyme research (Shanghai, China). LX-2 cells were cultured in DMEM-LG containing 10% FBS and 100 IU/mL penicillin/streptomycin. To investigate fibrotic molecules expression on activated HSCs, LX-2 cells grown in DMEM-LG presence of 10% FBS were starved for 24 h in medium containing 0.2% FBS and then cultured within TGF-β1 (Sigma, St. Louis, MO, USA) at a concentration of 10 ng/mL for 48 h.

### Co-culture system

To assess the effects of HUC-MSCs on activated HSCs, an indirect co-culture system in a transwell chamber (24 mm diameter, 0.4 µm pore size, Corning, NY, USA) was used. TGF-β1-activated LX-2 cells were co-cultured with HUC-MSCs in DMEM-LG containing 10% FBS at a ratio of 1:1 performed with LX-2 cells inoculated in the lower part and HUC-MSCs inoculated in the upper part. Compared with co-culture group, activated LX-2 cells were cultured alone in the lower part without cells inoculated in the upper part. LX-2 cells were collected and processed after culturing for 48 h.

### MicroRNA high-throughput sequencing

Total RNAs were extracted from LX-2 cells (cultured with or without HUC-MSCs), respectively, using TRIzol reagent (Invitrogen, Carlsbad, CA, USA) and purified using mirVana miRNA Isolation Kit (Life Technologies, Grand Island, NY, USA). High-throughput sequencing of miRNA was performed by Cloudseq Biotechnology Co., Ltd (Shanghai, China) sequenced on an Illumina HiSeq 2000 platform (Illumina, Inc., San Diego, CA, USA).

### Quantitative real-time PCR

Total RNA was isolated from LX-2 cells, mouse liver tissues, and clinical samples using TRIzol reagent according to the manufacturer’s protocol. To quantify mRNA expression, total RNA was reverse transcribed into cDNA using the PrimeScript RT reagent kit (Takara, Shanghai, China). Quantitative real-time PCR (qRT-PCR) was carried out using SYBR Premix Ex Taq (Takara). To measure mature miRNAs expression, reverse transcription was performed using TaqMan MicroRNA Reverse Transcription Kit, and the quantification of mature miRNAs was tested using the TaqMan miRNA assay system (Life Technologies). Relative expression of target genes and miRNAs was calculated by the 2^−ΔΔCt^ method. GAPDH and U6 were used as reference genes. The gene-specific primers are listed in Additional file 1: Table 1.

### Western blot

Total protein was extracted from LX-2 cells, mouse, and human liver tissues by lysing in RIPA buffer (Millipore, Billerica, MA, USA) supplemented with protease inhibitors (1% phenylmethanesulfonyl fluoride (PMSF)). The protein concentration was quantified by the BCA protein assay kit (Beyotime, Nanjing, China) using BSA as standard. Total protein was separated by loading on 6%-10% Trisglycine SDS-PAGE and then transferred onto polyvinylidene fluoride (PVDF) membranes (Millipore, Darmstadt, Germany). Primary antibodies incubated on the membranes used in this study were as follows: α-SMA(1:500; Affinity, Changzhou, China), Col1α1 (1:1000; Abcam, Cambridge, MA, USA), Notch2 (1:1000; Cell Signaling Technology, Beverly, MA, USA), Notch3 (1:1000; Abcam), Hes1 (1:1000; Abcam), β-actin (1:1000; Abcam), GAPDH (1:2500; Abcam), and Vinculin (1:1000; Abcam). The expression levels of β-actin, GAPDH, or Vinculin were used as the control for total protein amount. Horseradish peroxidase (HRP)-conjugated goat anti-rabbit immunoglobulin G (IgG) (1:1000; Cell Signaling Technology) was used as a secondary antibody at room temperature for 1 h. Protein bands were detected using the chemiluminescence HRP substrate (Millipore). The densitometric analysis of immunoblots was measured by Gel-Pro Analyzer software (Media Cybernetics, Rockville, MD, USA).

### Cell transfection

LX-2 cells at 70% confluency were transfected with 50 nM miR-154-5p or miR-148a-5p mimics, inhibitors, or their negative controls (NC) using Lipofectamine 3000 (Invitrogen) in Opti-MEM (Invitrogen) according to the manufacturer’s instructions. The synthetic miR-154-5p and miR-148a-5p mimics, inhibitors, mimics control, and inhibitors control were purchased from GenePharma (Shanghai, China). After transfection, cells were maintained in an incubator at 37 °C in a humidified atmosphere containing 5% CO_2_ for 24 h.

### Dual‑luciferase reporter assay

The pmirGLO luciferase reporter plasmid for 3’ UTR-WT (wild-type) or 3’ UTR-MUT (mutant, with a mutation in the has-miR-148a-5p binding core) of Notch2 was constructed. For luciferase assays, LX-2 cells were seeded into 24-well plates; after the confluence reaches 70–80%, the cells were cotransfected with pmirGLO/Notch2 3’ UTR-WT plasmid or 3’ UTR-MUT plasmid and miR-148a-5p mimics or negative controls (NC) using Lipofectamine 3000 (Invitrogen). Forty-eight hours after transfection, the cells were harvested and assayed for luciferase reporter activity using the Dual-Luciferase Reporter Assay System (Promega, Madison, WI, USA).

### Animals and treatments

Animal protocols were carried out according to the guidelines of Jiangsu University Institutional Animal Care and Use Committee (Zhenjiang, China). Seventy-five BALB/c male mice aged at 8 weeks were obtained from Laboratory Animal Center of Jiangsu University (Zhenjiang, China), weighing about 22–25 g. Mice were randomly divided into three groups: sham operation group (sham), Bile duct ligation group (BDL), and BDL + MSC group with 25 animals each. Mice underwent sham or BDL operation were anesthetized by intraperitoneal injection of 5% chloral hydrate. Briefly, the common bile duct was exposed through a midline abdominal incision. The BDL operation was performed by double ligation of the common bile duct with 5–0 sutures, while the sham operation was only performed the separation of the common bile duct without ligation. For mice treatment, in the sham and BDL groups, mice were injected with PBS through portal vein during the operation, while mice in BDL + MSC group were injected with 1 × 10^6^ HUC-MSCs. Ten mice in each group were killed on the 14th day after operation to obtain blood, liver tissue, or hepatocytes for further analyses, and the remaining 15 mice were used to evaluate the survival time.

### Primary hepatocytes isolation

Mouse primary hepatocytes were isolated by a three-step collagenase perfusion method as previous research [30]. Briefly, mouse was anesthetized and made abdominal incision to fully expose the portal vein and inferior vena cava. Liver was perfused sequentially by the following solutions via the inferior vena cava at a rate of 5 mL/min for 5 min: perfusion buffer (HBSS without calcium or magnesium, containing EGTA and HEPES) and digestion buffer (HBSS with calcium and magnesium, containing HEPES and collagenase IV). The digested liver tissue was removed and disrupted in cold William’s medium E, then, was filtered with a mesh filter, and washed three times by centrifugation at 50 g for 2 min at 4 ℃. Finally, the isolated cells were pelleted by centrifugation at 50 g for 10 min at 4 ℃ for qRT-PCR analysis or seeded and cultured in hepatocyte culture medium at 37 ℃ in 5% CO_2_ for microscopic observation.

### Histology, immunohistochemistry, and TUNEL staining

Liver tissues were fixed in 10% neutral buffered formalin and then embedded in paraffin for further histopathological analysis. Sections were cut into 4-μm thickness and stained standard protocols with hematoxylin and eosin (H&E) to examine morphology, stained Masson, and Sirius red to detect fibrotic processes. For immunohistochemistry (IHC) analysis, the liver sections were dewaxed in xylene and then dehydrated in a descending alcohol series. Heat-induced antigen retrieval was performed by incubation in citrate buffer (pH 6.0) for 15 min. The sections were subsequently incubated in 2% bovine serum albumin for 30 min to block nonspecific binding sites. Next, the slides were incubated with primary antibody specific for α-SMA (1:200; Affinity), Hes1 (1:100; Abcam), Notch2 (1:250; Cell Signaling Technology), Notch3 (1:250; Abcam), or Ki-67(1:200; Affinity) overnight at 4 °C. Then, the slides were treated with secondary antibody (1:500; HRP-conjugated anti-rabbit IgG, Cell Signaling Technology) for 1 h at room temperature. Subsequently, the reaction products were visualized by covering with diaminobenzidine (DAB) and counterstained with hematoxylin. For TUNEL staining, the paraffin sections of liver tissue were dewaxed and dehydrated as described by IHC analysis. Then, the experimental steps were carried out according to the instructions of one-step TUNEL cell apoptosis detection kit (green fluorescence) (Beyotime, Shanghai, China). The images were captured using a fluorescence microscope (OlympusBX-53, Tokyo, Japan).

### Biochemical analysis

Serum alanine aminotransferase (ALT), aspartate aminotransferase (AST), total protein (TP), and total bilirubin (TBIL) were assessed using the Automated Biochemical Analyzer (AU-680, Beckman, Germany). Hydroxyproline content in liver tissue was detected using a kit (Nanjing Jian cheng Bioengineering Institute, Nanjing, China) according to the manufacturer’s instructions.

### Human specimens

Human liver tissues were provided by the Affiliated Hospital of Jiangsu University (Zhenjiang, China) from patients who had undergone partial hepatectomy and signed the specimens obtaining informed consent. The tissues were quickly frozen in liquid nitrogen after surgical resection. Samples of patients with different stages of liver fibrosis were analyzed according to Brunt scoring systems [31]. Finally, 19 cases of healthy liver and 16 cases of liver fibrosis were used for analysis. All procedures were approved by the Medical Ethics Committee of Jiangsu University.

### Statistical analysis

Experimental results are expressed as mean ± SD. Statistical analysis was performed with SPSS 20.0 software (SPSS, Inc., Chicago, IL, USA). Statistical differences were analyzed by either the Student’s t test for comparison of two groups or one-way analysis of variance (ANOVA) for comparison among multiple groups. Survival curves were analyzed using the Kaplan–Meier method and compared with the log-rank test. Differences with *P* < 0.05 were considered statistically significant.

## Results

### MiR-148-5p is up-regulated by HUC-MSCs in HSCs and inhibits the activation of HSCs

**Fig. 1:**
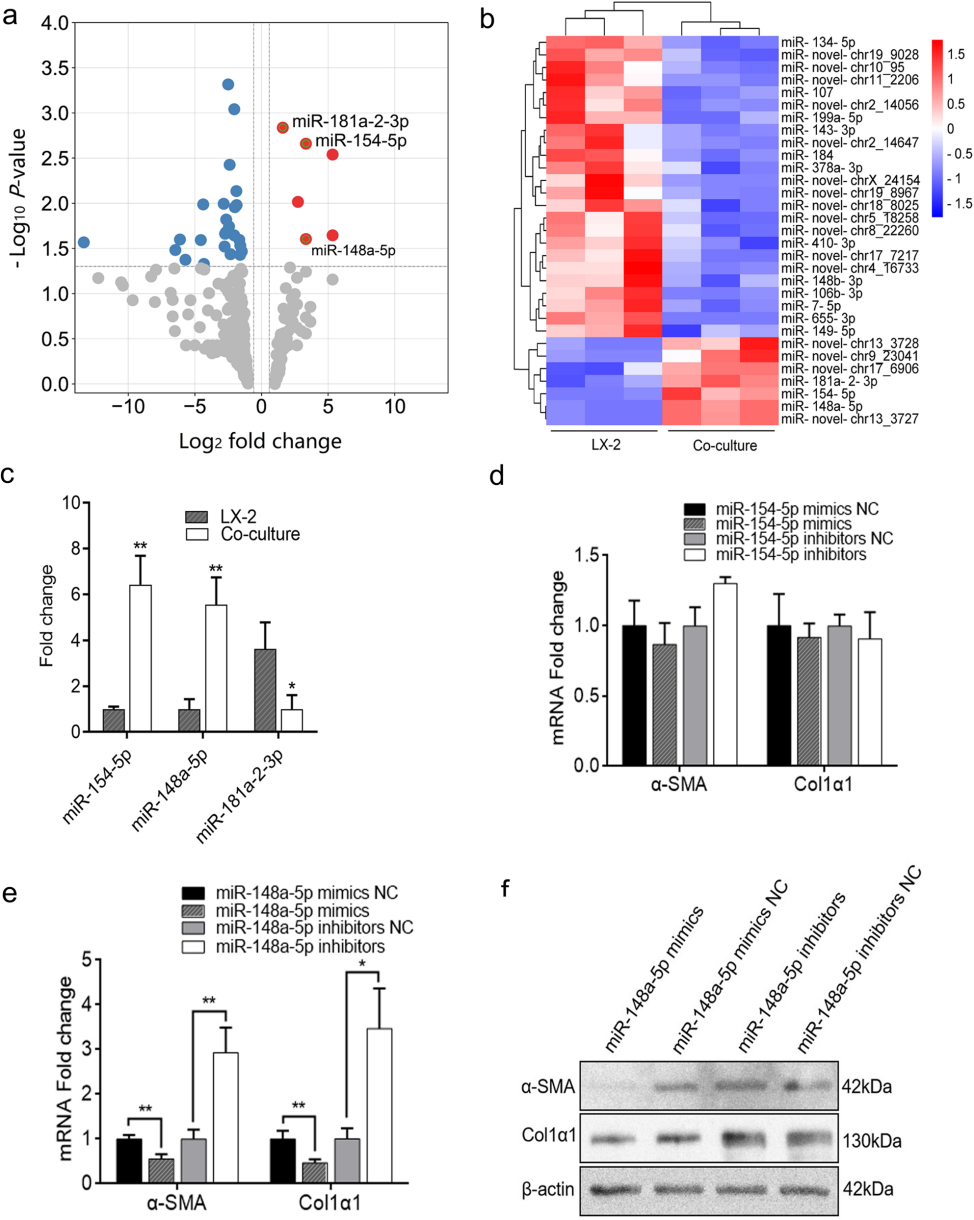
MiR-148-5p is up-regulated by HUC-MSCs in HSCs and inhibits the activation of HSCs. **a** Volcano plot of differentially expressed miRNAs in activated LX-2 cells cultured with or without HUC-MSCs from high-throughput sequencing. **b** Heatmap of differentially expressed miRNAs in activated LX-2 cells cultured with or without HUC-MSCs. **c** Three significantly up-regulated miRNAs were verified by qRT-PCR. **d, e** The mRNA levels of α-SMA and Col1α1 were assessed by qRT-PCR in activated LX-2 cells transfected with miR-154-5p mimics, miR-154-5p inhibitors, miR-148a-5p mimics, 148a-5p inhibitors, or their negative controls, respectively. **f** The protein levels of α-SMA and Col1α1 were detected by Western blot in LX-2 cells transfected with miR-148a-5p mimics, 148a-5p inhibitors, or their negative controls. The quantitative data are represented as the mean ± SD. Each experiment was repeated three times. *P < 0.05 and **P < 0.01. *HSCs* hepatic stellate cells; *HUC-MSCs* human umbilical cord mesenchymal stem cells; *miRNA* microRNAs; *qRT-PCR* quantitative real-time PCR; *α-SMA* α-smooth muscle actin; *Col1α1* collagen type I α1

In our previous study, we co-cultured TGF-β1-activated human HSC cell line LX-2 cells with HUC-MSCs by a transwell indirect co-culture system and found that HUC-MSCs can inhibit HSCs activation by inhibiting the expression of α-SMA and Col1α1 compared with the LX-2 cells cultured alone group [32]. To assess whether HUC-MSCs inhibit HSCs activation by affecting the expressions of miRNAs, we performed miRNAs high-throughput sequencing on total RNAs extracted from TGF-β1-activated LX-2 cells co-cultured with or without HUC-MSCs. In comparison with the single culture group, 34 miRNAs were significantly differentially expressed after HUC-MSCs treatment (fold change > 1.5, *P* < 0.05), and 16 of them were novel miRNAs (Fig. 1a, b). We were interested in the up-regulated miRNAs and selected the three mature miRNAs including miR-181a-2-3p, miR-154-5p, and miR-148a-5p for further investigation and then validated these miRNAs in co-culture experiments by qRT-PCR. Our results showed that the expressions of miR-154-5p and miR-148a-5p were up-regulated in accordance with the high-throughput sequencing analysis (Fig. 1c). However, the expression of miR-181a-2-3p was down-regulated in the co-culture group (Fig. 1c). Therefore, miR-154-5p and miR-148a-5p were selected for further study. To confirm whether these two miRNAs were associated with liver fibrosis, TGF-β1-activated LX-2 cells were transfected with miR-154-5p or miR-148a-5p mimics/inhibitors, or their negative control (NC), respectively. We observed that the expressions of α-SMA and Col1α1 in cells transfected with miR-148a-5p mimics were significantly down-regulated, while in cells transfected with miR-148a-5p inhibitors which were significantly up-regulated by qRT-PCR and Western blot analysis (Fig. 1e, f). However, no significantly different expressions of α-SMA and Col1α1 were found after miR-154-5p transfection (Fig. 1d). Therefore, miR-148a-5p was selected as the miRNA for further study. In conclusion, our results indicated that HUC-MSCs inhibited the activation of HSCs by up-regulating the expression of miR-148a-5p.

### HUC-MSCs up-regulate the expression of miR-148a-5p in HSCs by suppressing the Notch signaling pathway

**Fig. 2:**
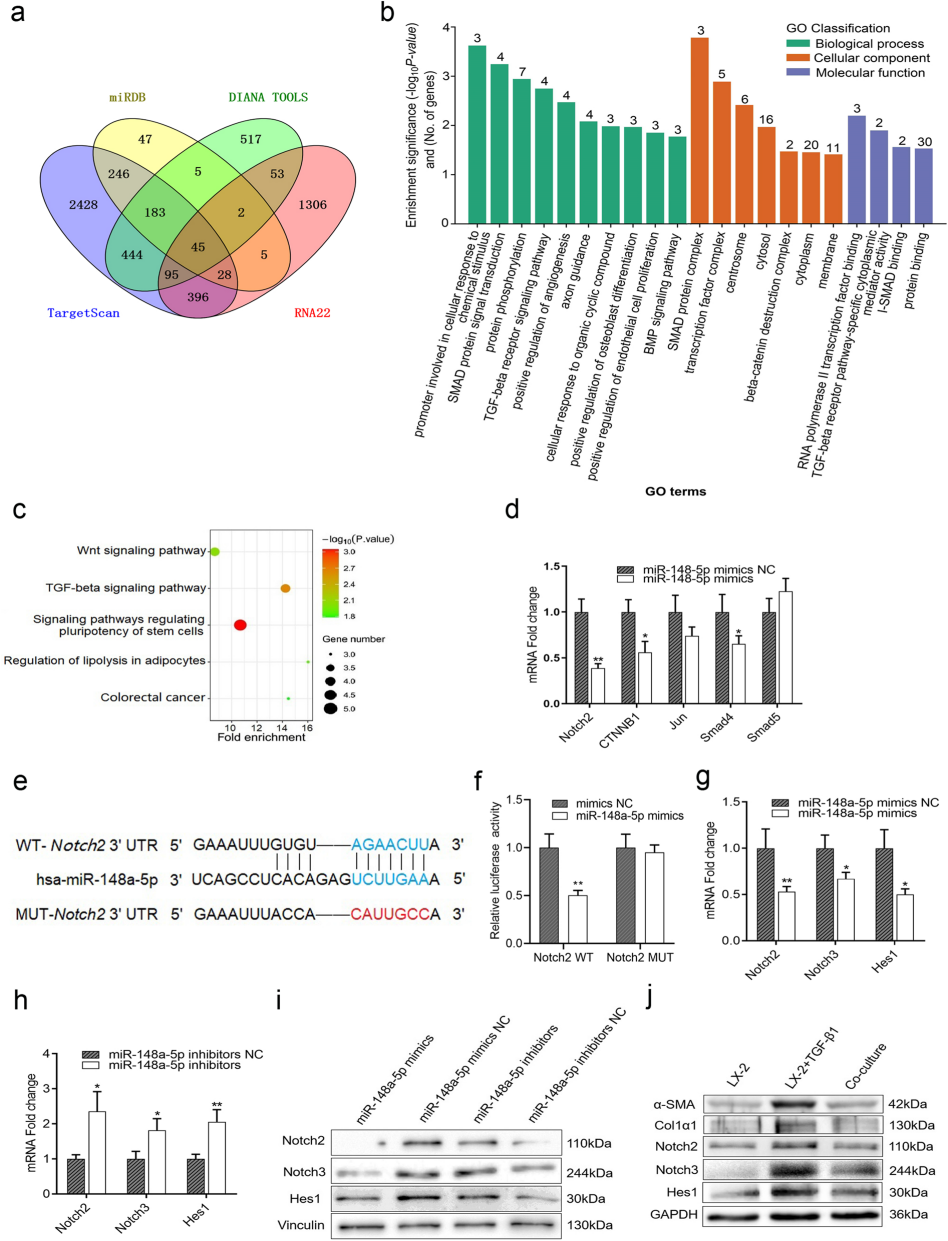
HUC-MSCs up-regulate the expression of miR-148a-5p of HSCs by suppressing Notch signaling pathway. **a** Venn diagram of overlapping target genes of miR-148a-5p predicted by multiple bioinformatics websites (TargetScan, miRanda, miRDB, and DIANA-microT). **b** GO enrichment results showing differences in target genes based on their distribution by GO terms. **c** KEGG analysis of candidate genes. **d** Five fibrosis-related predictive target genes of miR-148a-5p were verified by qRT-PCR in LX-2 cells transfected with miR-148a-5p mimics or NC. **e** Schematic representation of miR-148a-5p binding to predicted recognition sites in the 3’ UTR of Notch2. **f** Luciferase activity assay on LX-2 cells cotransfected with Notch2 WT 3’ UTR-luciferase plasmid or Notch2 MUT 3’ UTR-luciferase plasmid and miR-148a-5p mimics or miR-148a-5p NC. **g, h** The mRNA levels of Notch2, Notch3, and Hes1 were detected by qRT-PCR in LX-2 cells transfected with miR-148a-5p mimics, 148a-5p inhibitors, or their negative controls, respectively. **i** The protein levels of Nocth2, Notch3, and Hes1 were detected by Western blot in LX-2 cells transfected with miR-148a-5p mimics, inhibitors, or their NC, respectively. **j** The protein levels of α-SMA, Col1α1, Nocth2, Notch3, and Hes1 were detected in LX-2 cells treated with or without TGF-β1 cultured alone or co-cultured with HUC-MSCs. The quantitative data are represented as the mean ± SD. Each experiment was repeated three times. *P < 0.05 and **P < 0.01. *HUC-MSCs* human umbilical cord mesenchymal stem cells; *HSCs* hepatic stellate cells; *GO* gene ontology; *KEGG* Kyoto Encyclopedia of Genes and Genomes; *MUT* mutant; *qRT-PCR* quantitative real-time PCR; *NC* negative control; *TGF-β1* transforming growth factor-β1; *WT* wild-type

To identify the molecular mechanism of miR-148a-5p regulates HSCs activation, we performed bioinformatics analyses with web-based tools including miRDB, DIANA TOOLS, TargetScan, and RNA22 and screened out 45 potential target genes of miR-148a-5p (Fig. 2a). Gene Ontology (GO) analysis was performed to evaluate mRNA enrichments in terms of biological process, cellular component, and molecular function. The biological processes of these target genes were mainly enrichment in the SMAD protein signal transduction and the TGF-β receptor signaling pathway, and the molecular functions were mainly enriched in the TGF-β receptor pathway-specific cytoplasmic mediator activity and the I-SMAD binding, which were well known to be associated with hepatic fibrosis (Fig. 2b). The Kyoto Encyclopedia of Genes and Genomes (KEGG) analysis revealed 5 pathways, including the Wnt signaling pathway and the TGF-β signaling pathway (Fig. 2c), which were known to be involved in the progression of liver fibrosis. Among them, Smad4, Jun, Csnk1a1, and Ctnnb1 were potential target genes of miR-148a-5p in the Wnt signaling pathway, and Smad4, ID4, Smad9, and Smad5 were potential target genes in the TGF-β signaling pathway. Since the genes of Smad4, Jun, Ctnnb1, and Smad5 have been reported to be associated with liver fibrosis, and the Notch2 gene was considered to be related to Wnt and TGF-β signaling pathways, these five candidate target genes were selected for research. To clarify the targeting relationship between these genes and miR-148a-5p, TGF-β1-activated HSCs were transfected with miR-148a-5p mimics or NC; the result showed that Notch2 was decreased most significantly in the five-candidate target genes after transfected with miR-148a-5p mimics (Fig. 2d). Then, the relationship of Notch2 directly targeted by miR-148a-5p was validated by luciferase assay on wild-type and mutagenized 3’ UTR from Notch2 (Fig. 2e, f). To investigate whether miR-148a-5p affects HSCs activation through Notch signaling pathway, Notch signaling-related key nodes and target genes Notch2, Notch3, and Hes1 were detected in TGF-β1-activated HSCs transfected with miR-148a-5p mimics/inhibitors or these NC. The results showed that mRNA and protein levels of Notch2, Notch3, and Hes1 were decreased in HSCs after transfection of miR-148a-5p mimics, whereas increased after transfection of miR-148a-5p inhibitors (Fig. 2g–i). To confirm whether HUC-MSCs inhibit HSCs activation by inhibiting Notch signaling pathway, TGF-β1-activated HSCs were co-cultured with HUC-MSCs. We found that the levels of α-SMA and Col1α1 in HSCs were decreased after co-cultured with HUC-MSCs, which indicated a reduction in the activity of HSCs (Fig. 2j). Moreover, the protein levels of Notch2, Notch3, and Hes1 were decreased at the same time (Fig. 2j). Taken together, our data suggested that HUC-MSCs inhibit HSCs activation by up-regulating the expression of miR-148a-5p through inhibiting the Notch signaling pathway.

### HUC-MSCs ameliorate BDL-induced liver fibrosis in mice

**Fig. 3:**
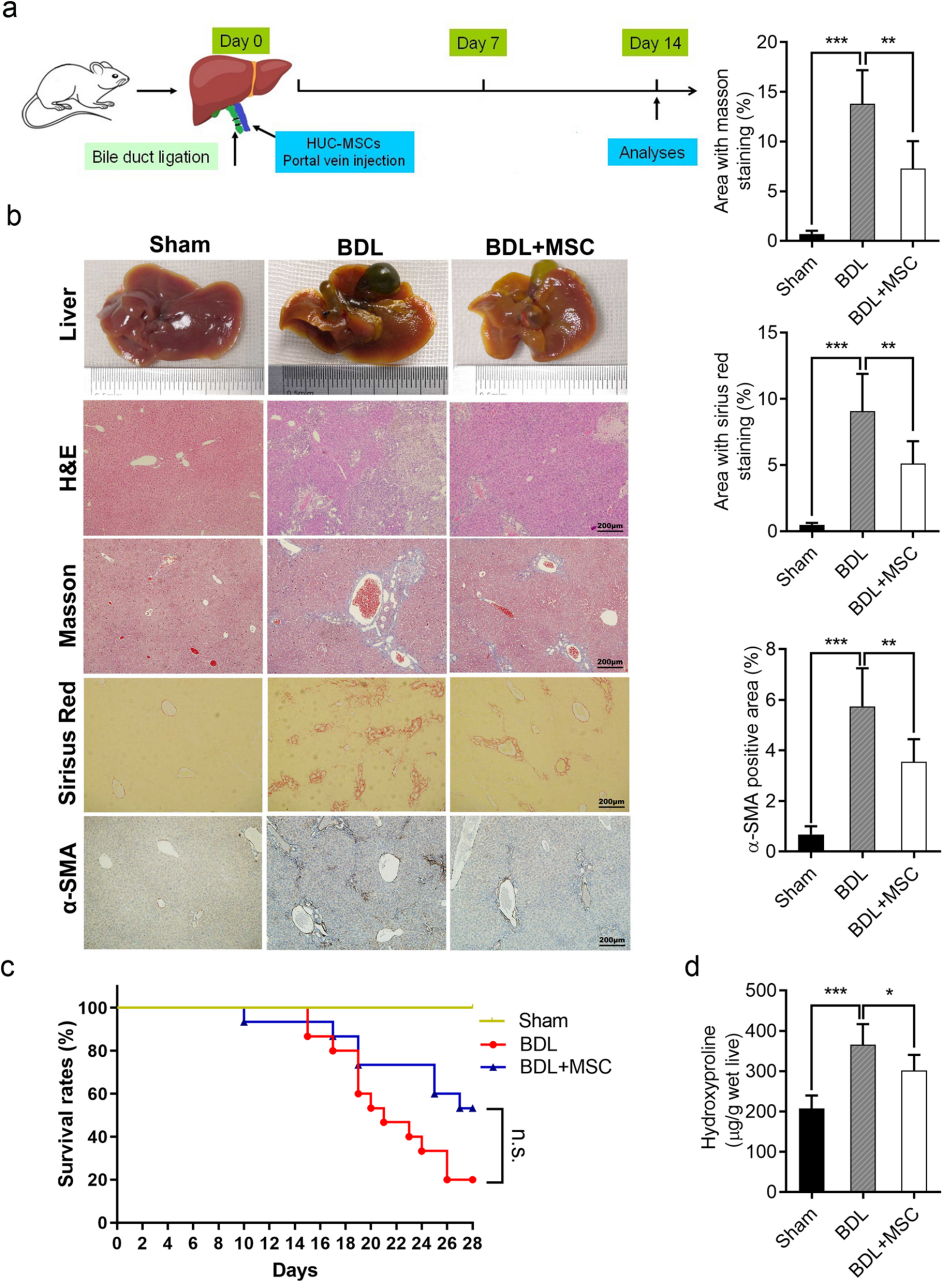
HUC-MSCs ameliorate BDL-induced liver fibrosis in mice. **a** Schema depicting HUC-MSCs treatment for BDL-induced liver fibrosis. **b** Representative images of liver gross morphology of sham, BDL, and BDL + MSC groups, and images of H&E, Masson, Sirius red, and α-SMA immunohistochemistry staining of liver sections of the three groups. Scale bars, 200 µm. **c** Kaplan–Meier survival analysis of mice in the three groups. **d** The amount of liver hydroxyproline was detected in the different groups. Each experiment was repeated three times. *P < 0.05, **P < 0.01, ***P < 0.001 and n.s. not significant. *HUC-MSCs* human umbilical cord mesenchymal stem cells; *α-SMA* α-smooth muscle actin

To further investigate the role of HUC-MSCs in hepatic fibrosis in vivo, the mouse liver fibrosis model was established by bile duct ligation, HUC-MSCs were injected through the portal vein during the operation. Five mice each from sham, BDL, and BDL + MSC groups were killed for various microscopic evaluations 14 days after the operation (Fig. 3a). To examine whether HUC-MSCs ameliorate BDL-induced liver fibrosis, macroscopic examination, hematoxylin and eosin (H&E), Masson and Sirius red staining were used to evaluate the extent of liver fibrosis. Macroscopic examination showed that livers in the BDL + MSC group were smoother and softer than those in BDL group (Fig. 3b). Besides, H&E-stained liver sections from BDL group revealed extensive hepatocellular death with cytoplasmic vacuolization and severe structural destruction of liver tissue (Fig. 3b). By contrast, liver sections from HUC-MSCs-treated group rarely showed hepatocellular edema and death. Moreover, Masson and Sirius red staining all indicated less collagen deposition and smaller fibrotic areas after HUC-MSCs transplantation (Fig. 3b).

α-SMA is regarded as a marker of myofibroblasts and reflects the severity of liver fibrosis. The result of IHC for α-SMA showed administration of HUC-MSCs greatly reduced liver fibrosis by reducing the area of a-SMA staining (Fig. 3b). In addition, the hepatic hydroxyproline contents in BDL + MSC group were considerably lower in comparison with the BDL group (Fig. 3d).

During the 28-day follow-up period after sham or BDL operation, mice in sham group were all survival, and 12 of the 15 mice in BDL group were died in succession. By contrast, better survival rate was observed for HUC-MSCs treatment, with only 7 mice dead during the observation period (Fig. 3c). Overall, these results suggested that HUC-MSCs injection could attenuate BDL-induced liver fibrosis in mice.

### HUC-MSCs inhibit Notch signaling pathway by up-regulating miR-148-5p in BDL-induced liver fibrosis

**Fig. 4:**
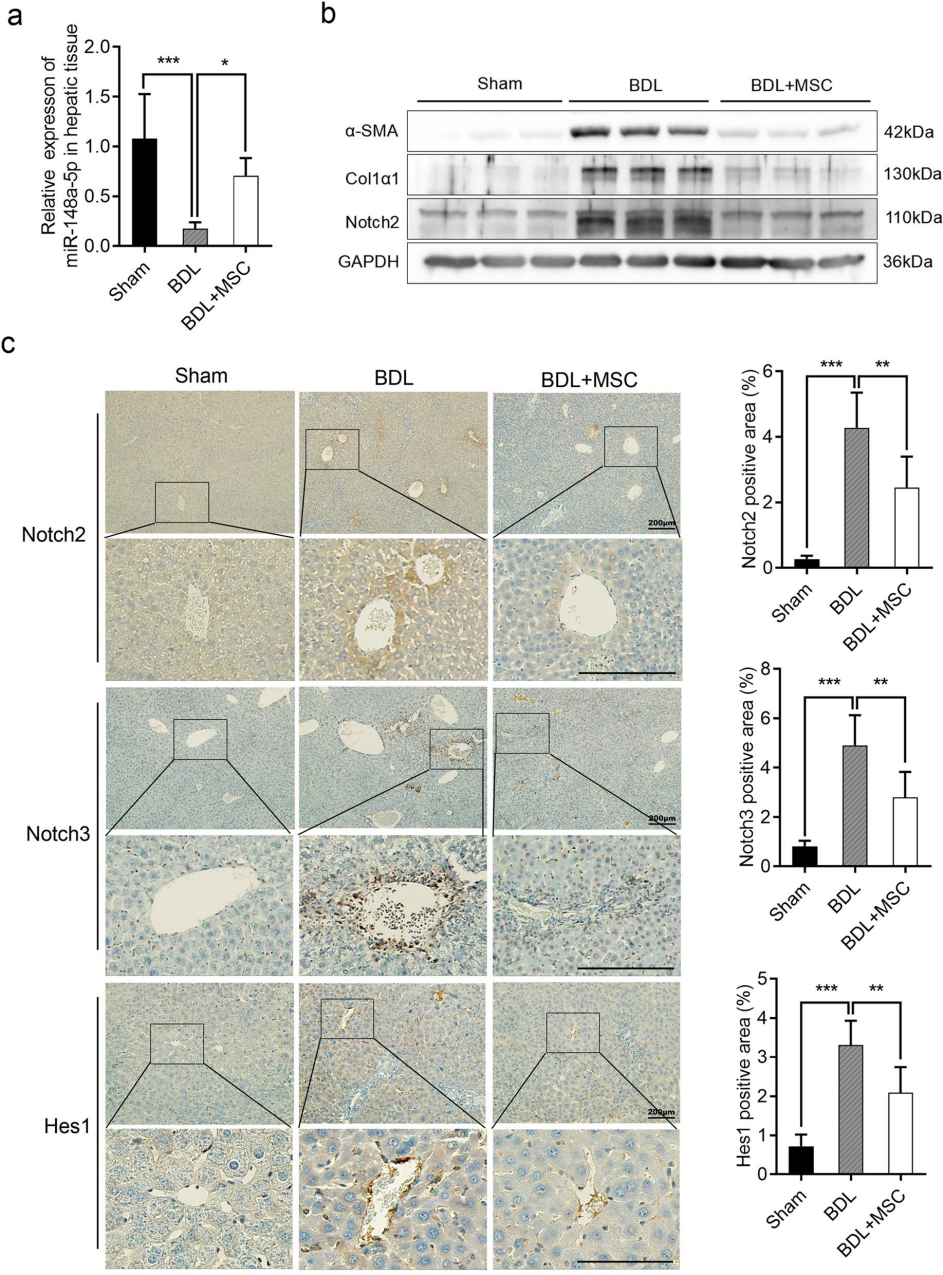
HUC-MSCs inhibit Notch signaling pathway by up-regulating miR-148-5p in BDL-induced liver fibrosis. **a** The expression levels of miR-148-5p were examined in mouse hepatic tissues of sham, BDL, and BDL + MSC groups. **b** The protein levels of α-SMA, Col1α1, and Notch2 in mouse hepatic tissues were detected by Western blot. **c** Immunohistochemical staining of Notch2, Notch3 and Hes1 in liver tissues of different groups, and quantification of Notch2, Notch3, and Hes1-positive areas in mouse hepatic tissues. Scale bars, 200 µm. The quantitative data are represented as the mean ± SD. Each experiment was repeated three times. *P < 0.05, **P < 0.01, and ***P < 0.001. *HUC-MSCs* human umbilical cord mesenchymal stem cells; *α-SMA* α-smooth muscle actin; *Col1α1* collagen type I α1

To investigate whether miR-148-5p is involved in the therapeutic capacity of HUC-MSCs in BDL-induced liver fibrosis, the expression of miR-148-5p in mouse livers was detected by qRT-PCR. The results showed that miR-148-5p was significantly up-regulated after HUC-MSCs treatment compared with BDL model group (Fig. 4a). To further identify the function of miR-148-5p in fibrotic tissue, the protein level of Notch2, a target gene of miR-148-5p, which has been confirmed before was analyzed by Western blotting. Consistent with experiments in vitro, with the decrease of the profibrotic markers including α-SMA and Col1α1, a significantly lower expression level of Notch2 was found in hepatic fibrosis mice transplanted with HUC-MSCs compared with the BDL control group (Fig. 4b). To investigate whether HUC-MSCs attenuate liver fibrosis by suppression of Notch signaling pathway as the findings in vitro, Notch signaling-related molecules including Notch2, Notch3, and Hes1 were examined by immunochemistry. As we expected, their expressions were significantly decreased after HUC-MSCs treatment (Fig. 4c). Taken together, these results indicated that HUC-MSCs alleviated hepatic fibrosis through up-regulating miR-148-5p by targeting Notch2 and suppressing the Notch signaling pathway.

### HUC-MSCs inhibit hepatocellular apoptosis and enhance liver regeneration by up-regulating miR-148-5p in mice

**Fig. 5:**
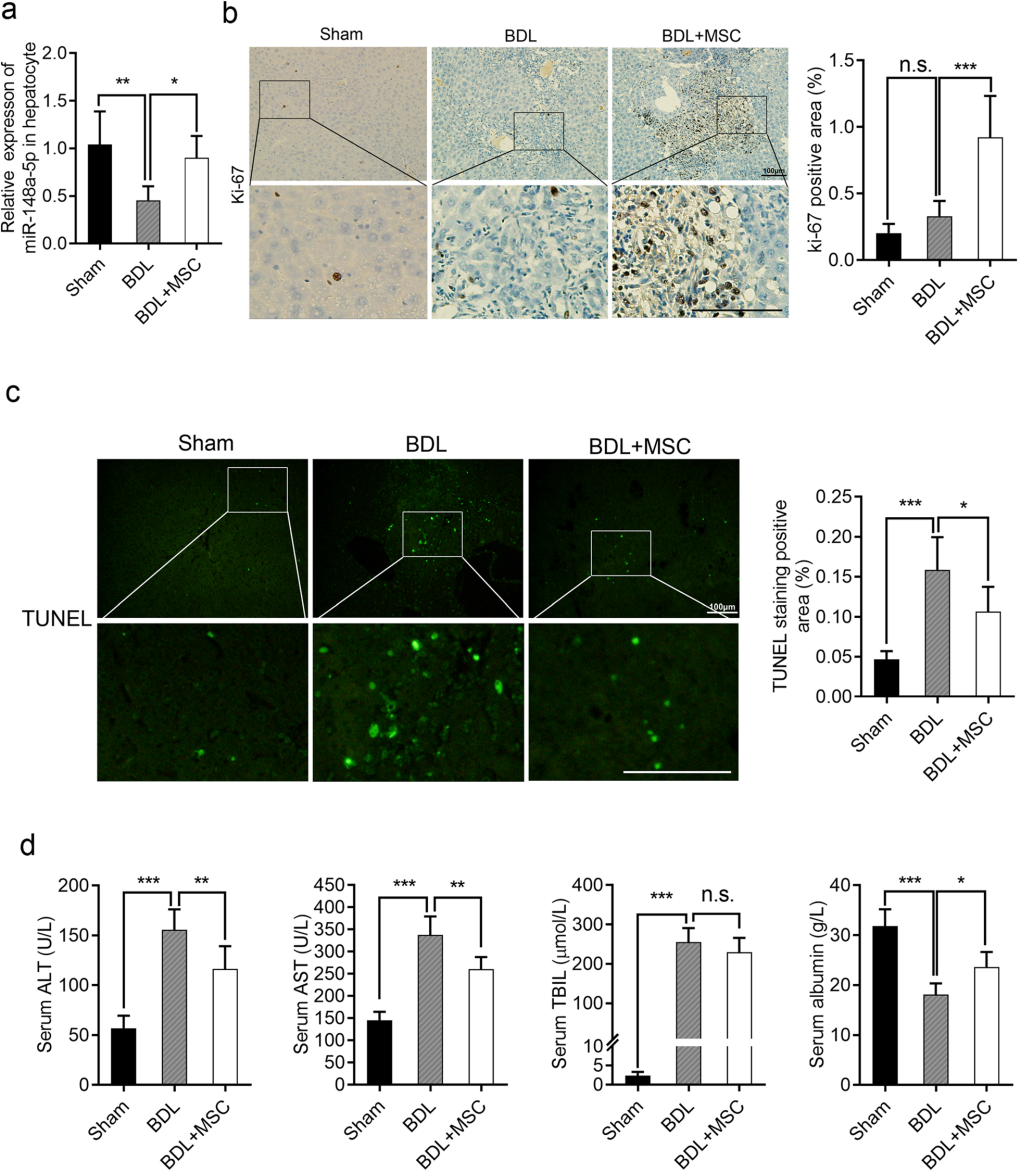
HUC-MSCs inhibit hepatocellular apoptosis and enhance liver regeneration by up-regulating miR-148-5p in mice. **a** The expression levels of miR-148-5p in hepatocytes extracted from mouse hepatic tissues of sham, BDL, and BDL + MSC groups. **b** Immunohistochemical staining of Ki-67 in liver tissues of the three groups. **c** TUNEL staining of liver sections from the three groups. **d** Serum levels of ALT, AST, TBIL, and albumin in the three groups. Each experiment was repeated three times. *P < 0.05, **P < 0.01, ***P < 0.001 and n.s. not significant. *HUC-MSCs* human umbilical cord mesenchymal stem cells; *ALT* alanine aminotransferase; *AST* aspartate aminotransferase; *TBIL* total bilirubin

Hepatocyte apoptosis and regeneration play an important role in liver fibrosis. To clarify the role of miR-148-5p in hepatocytes, primary hepatocytes were extracted from mice underwent sham or BDL operation. Compared with mice in sham group, miR-148-5p expression was significantly decreased in primary hepatocytes of mice underwent BDL, while HUC-MSCs treatment up-regulated the expression of miR-148-5p (Fig. 5a). To determine the effect of HUC-MSCs on hepatocyte proliferation, the proliferation marker Ki-67 was used to detect the proliferation of hepatocytes in liver tissue sections of each group. Few Ki-67-positive hepatocytes were observed in livers of sham and BDL groups, whereas significantly increased positive cells were observed in HUC-MSCs-treated liver (Fig. 5b).

Hepatocyte apoptosis is one of the factors leading to liver fiber. To determine whether HUC-MSCs transplantation affects hepatocyte apoptosis, hepatocyte nuclei in liver sections were detected by TUNEL staining. Many TUNEL-reactive hepatocyte nuclei were observed in control mice with BDL, yet dramatic reduction of apoptotic hepatocyte nuclei was observed in sham and HUC-MSCs-treated mice (Fig. 5c).

Hepatocyte apoptosis and liver necrosis induced damage to liver function. To further evaluate the effect of HUC-MSCs on liver function, the serum of mice in each group was used to detect the expression of AST, ALT, TBIL, and albumin. Reduced AST and ALT, and increased albumin levels were observed in BDL-induced fibrotic livers of mice treated with HUC-MSCs compared with those in mice of BDL model group (Fig. 5d). However, no significant change was observed in TBIL, indicating that jaundice did not improved by HUC-MSCs treatment (Fig. 5d). Thus, these observations suggested that HUC-MSCs improve liver regeneration and liver function, and inhibit hepatocellular apoptosis in mice with liver fibrosis.

### Expression of miR-148-5p and Notch signaling pathway in human fibrosis hepatic tissues

**Fig. 6:**
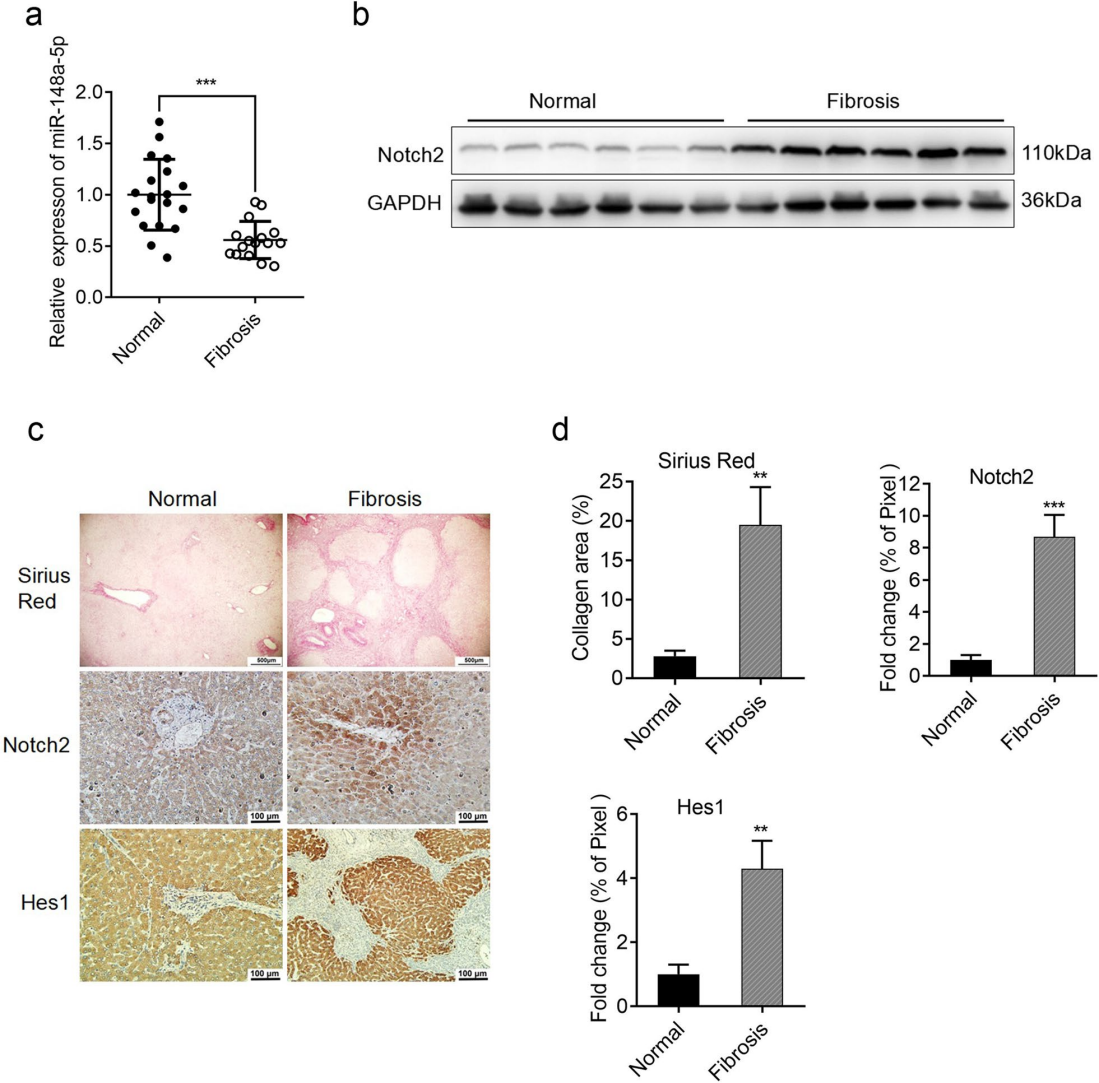
Expression of miR-148-5p and Notch signaling pathway in liver tissues of patients with fibrosis. **a** The expression levels of miR-148-5p were examined in normal and fibrosis liver tissues. **b** Western blot analysis showing the expression of Notch2 in normal and fibrosis liver tissues. Protein samples in different lanes were obtained from different individuals. **c** Histological analysis was performed by sirius red staining (Scale bars, 500 µm), and immunohistochemical analysis of normal and fibrosis liver tissues for the expression of Notch2 and Hes1 (Scale bars, 100 µm). **d** Collagen proportion (stained red) of sirius red staining, and quantification of Notch2 and Hes1-positive areas in normal and fibrosis liver tissues

To investigate whether miR-148-5p is involved in the process of human liver fibrosis, we detected the expression of miR-148-5p in normal liver and hepatitis B-induced liver fibrosis tissues. As the findings from experimental mice showed, the expression of miR-148-5p in human fibrotic tissues was significantly lower than that in normal tissues (Fig. 6a). The expression of Notch2 was also increased dramatically in fibrotic tissues detected by Western blot analysis and immunohistochemistry (Fig. 6b–d). Moreover, the expression of Hes1 was also significantly increased in fibrotic tissues compared with that in normal livers (Fig. 6c, d). These results indicated that the high expression of miR-148-5p and the low expression of Notch2 in liver tissue might predict the occurrence of liver fibrosis. Due to human ethical reasons, we have not yet applied MSCs for the treatment of human liver fibrosis, but miR-148-5p may be a potential target for MSCs treatment in human liver fibrosis.

## Discussion

Liver fibrosis, which is often characterized by progressive destruction of liver structure, chronic inflammatory infiltration, liver dysfunction, and portal hypertension [1], is considered as a significant public health issue and increases the risk of developing hepatocellular carcinoma [33]. Currently, there are no effective treatments for end-stage liver fibrosis except for liver transplantation [34]. Therefore, alternative therapeutic approaches are urgent to be invented to treat liver fibrosis.

MSCs have been shown to alleviate liver fibrosis in the experimental models and in recent clinical trials by inhibiting HSCs activation, reducing collagen deposition, and improving liver function [35]. Recently, increasing number of studies have shown that dysregulated expression of miRNAs on HSCs activation plays an essential role in the pathogenesis of liver fibrosis [21]. However, the underlying mechanisms to explain the effects of MSCs on liver fibrosis by affecting miRNA expression are still largely unknown. Here, we have identified that miR-148a-5p was the most significantly up-regulated after co-culture with HUC-MSCs in TGF-β1-activated HSCs using high-throughput sequencing.

MiR-148a was found in various human tissues, including heart, thymus, liver, pancreas, placenta, and renal [36]. It is considered as an oncogene or tumor suppressor gene in multiple types of tumors [26]. As a potent inducer of hepatocytic differentiation, miR-148a is conserved in humans and mice [23]. Several studies have indicated that miR-148a was highly expressed in normal liver but was lowly expressed in HCC and liver fibrous tissue [23, 37]. Down-regulation of miR-148a was often associated with poor TNM stage and low survival rate in patients with HCC [26, 38]. Moreover, miR-148a treatment shows a beneficial effect on liver fibrosis and liver function [23, 37]. However, most studies have focused on miR-148a-3p (the guide strand of the miR-148-family) [24, 39], and little is known regarding miR-148a-5p (the passenger strand) in cancer or fibrotic disease. In this study, we found that miR-148a-5p acted as an anti-fibrosis miRNA as role of miR-148-3p in hepatic fibrosis. In vitro, our results showed that miR-148a-5p was down-regulated in TGF-β1-activated LX-2 cells, while the expression of miR-148a-5p was significantly up-regulated after co-cultured with HUC-MSCs, accompanied by the decrease of proliferation ability and activity of LX-2 cells. In vivo, we observed that miR-148a-5p was down-regulated in BDL-induced mouse hepatic fibrosis, while HUC-MSCs treatment could up-regulate the expression of miR-148a-5p in the liver and alleviate liver fibrosis.

To determine the direct target gene of miR-148a-5p, we used bioinformatics analysis to predict and confirm that Notch2 was the target gene of miR-148a-5p. The Notch signaling network involves four Notch receptors (Notch 1 to 4) in mammals, and all four Notch proteins are expressed in both the epithelium and mesenchyme of the adult liver [40]. Recent studies have shown that the Notch signaling pathway is involved in the regulation of differentiation of myofibroblasts in liver fibrosis, and disruption of Notch signaling can attenuate liver fibrosis [41]. Expressions of Notch2, Notch3, and Notch target gene Hes1 were significantly up-regulated during the development of hepatic fibrosis [42]. Selective interruption of Hes1 could decrease the expression of α-SMA and Col1α2 and alleviate hepatic fibrosis [43]. Moreover, Notch antagonism with γ-secretase inhibitors could effectively reduce hepatic fibrosis in patients with nonalcoholic steatohepatitis (NASH) [44]. In this study, our data showed that TGF-β1 treatment could activate LX-2 cells and up-regulate Notch2, Notch3, and Hes1, while HUC-MSCs treatment could reverse their levels by increasing the expression of miR-148a-5p. Up-regulation of miR-148a-5p expression by transfecting miR-148a-5p mimics could inhibit the activation and reduce the expression of Notch2, Notch3, and Hes1 in LX-2 cells. In vivo, we also found that HUC-MSCs treatment suppresses the Notch pathways through the up-regulation of miR-148a-5p in BDL-induced liver fibrosis of mice.

MSC has been reported to improve liver function, inhibit hepatocyte apoptosis, and stimulate the regeneration of damaged liver cells [18, 45]. Our results were consistent with the previous reports, and these suggested that the protective effect of MSC on hepatocytes may be related to increasing the expression of miR-148a-5p in hepatocytes. In addition, we found that the expression level of miR-148a-5p in liver tissue of patients with cirrhosis was significantly decreased with the increase of Notch2 and Hes1 compared with the normal liver. In conclusion, we speculate that miR-148a-5p may play an anti-fibrogenic role not only in MSC-mediated anti-fibrosis, but also in endogenous regulation of liver fibrosis.

## Conclusion

**Fig. 7:**
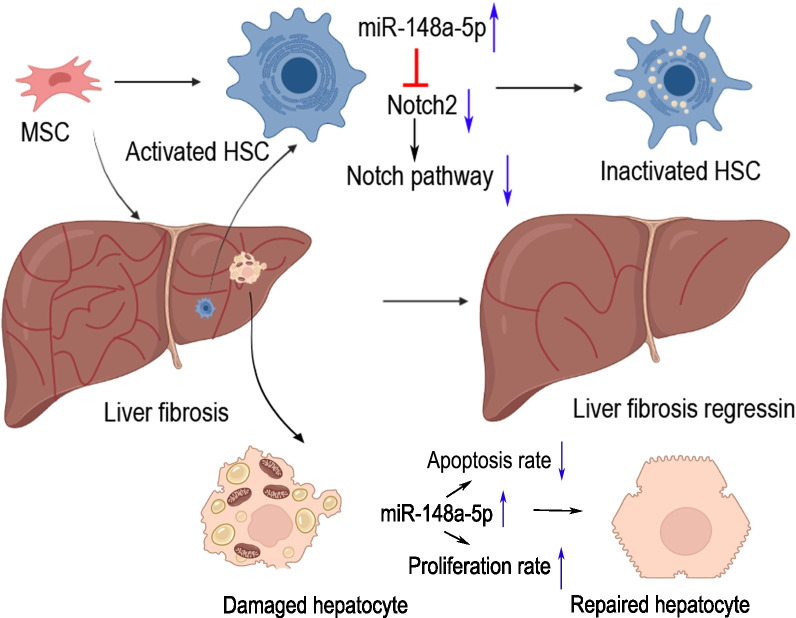
Schematic representation of MSCs inhibiting HSCs activation, protecting hepatocytes, and alleviating liver fibrosis by regulating miR-148a-5p/Notch2/Notch signaling pathway

These findings demonstrated that MSCs treatment significantly alleviates HSCs activation and BDL-induced liver fibrosis in mice. Overexpression of miR-148-5p by inhibiting the Notch signaling pathway might be the underlying mechanism for the therapeutical effect of MSCs in hepatic fibrosis (Fig. 7). MiR-148-5p has excellent potential to be used as a biomarker to monitor the progression of liver fibrosis.

## Supplementary Information

**Additional file 1**.** Supplementary Table 1**. Primers used in qRT-PCR analysis.

## Data Availability

MiRNA High-Throughput Sequencing data presented in the study can be obtained via the Gene Expression Omnibus under the accession codes GSE151098 (https://www.ncbi.nlm.nih.gov/geo/query/acc.cgi?acc=GSE151098). All other data are included in this published article and its supplementary information files.

## Abbreviations

ALT: Alanine aminotransferase
AST: Aspartate aminotransferase
BDL: Bile duct ligation
ECM: Extracellular matrix
FBS: Fetal bovine serum
GO: Gene ontology
HCC: Hepatocellular carcinoma
HSC: Hepatic stellate cell
HUC-MSC: Human umbilical cord mesenchymal stem cell
IHC: Immunohistochemistry
KEGG: Kyoto Encyclopedia of Genes and Genomes
miRNA: MicroRNA
MUT: Mutant
MSC: Mesenchymal stem cell
PBS: Phosphate-buffered saline
TB: Total protein
TBIL: Total bilirubin
TGF-β1: Transforming growth factor-β1
WT: Wild-type
