# Supplementary material for: Mesenchymal stem cells improve liver fibrosis and protect hepatocytes by promoting microRNA-148a-5p-mediated inhibition of Notch signaling pathway
Source: Stem Cell Res Ther. 2022 Jul 26;13:354. doi: 10.1186/s13287-022-03030-8 (PMC9327397; doi:10.1186/s13287-022-03030-8)
Supplement: Supplementary file 1 — Additional file 1. Supplementary Table 1. Primers used in qRT-PCR analysis. [file 13287_2022_3030_MOESM1_ESM.docx]

**Supplementary Table 1** Primers used in qRT-PCR analysis

| Gene | Primer sequence |
| --- | --- |
| α-SMA (human) | Forward: 5’-CTATGAGGGCTATGCCTTGCC-3’ |
|  | Reverse: 5’-GCTCAGCAGTAGTAACGAAGGA-3’ |
| Col1α1 (human) | Forward: 5’-GTGCGATGACGTGATCTGTGA-3’ |
|  | Reverse: 5’-CGGTGGTTTCTTGGTCGGT-3’ |
| Notch2 (human) | Forward: 5’-CCTTCCACTGTGAGTGTCTGA-3’ |
|  | Reverse: 5’-AGGTAGCATCATTCTGGCAGG-3’ |
| CTNNB1 (human) | Forward: 5’-CATCTACACAGTTTGATGCTGCT-3’ |
|  | Reverse: 5’-GCAGTTTTGTCAGTTCAGGGA-3’ |
| Jun (human) | Forward: 5’-TCCAAGTGCCGAAAAAGGAAG-3’ |
|  | Reverse: 5’-CGAGTTCTGAGCTTTCAAGGT-3’ |
| Smad4 (human) | Forward: 5’-CTCATGTGATCTATGCCCGTC-3’ |
|  | Reverse: 5’-AGGTGATACAACTCGTTCGTAGT-3’ |
| Smad5 (human) | Forward: 5’-TCTCCAAACAGCCCTTATCCC-3’ |
|  | Reverse: 5’-GCAGGAGGAGGCGTATCAG-3’ |
| Notch3 (human) | Forward: 5’-TGGCGACCTCACTTACGACT-3’ |
|  | Reverse: 5’-CACTGGCAGTTATAGGTGTTGAC-3’ |
| Hes1 (human) | Forward: 5’-TCAACACGACACCGGATAAAC-3’ |
|  | Reverse: 5’-GCCGCGAGCTATCTTTCTTCA-3’ |
| GAPDH (human) | Forward: 5’-GGAGCGAGATCCCTCCAAAAT-3’ |
|  | Reverse: 5’-GGCTGTTGTCATACTTCTCATGG-3’ |
| miR-154-5p | Forward: 5’-GCGCGTAGGTTATCCGTGTTG-3’ |
| miR-148a-5p | Forward: 5’-GCGCGAAAGTTCTGAGACACT-3’ |
| miR-181a-2-3p | Forward: 5’- GCGACCACTGACCGTTGAC-3’ |
| Common reverse sequence | Reverse: 5’-GTGCAGGGTCCGAGGTATTC-3’ |
| U6 | Forward: 5’-CTCGCTTCGGCAGCACATATACT-3’ |
|  | Reverse: 5’-ACGCTTCACGAATTTGCGTGTC-3’ |
